# Transportation Use And Accessibility Among Persons with Dementia who Reside Alone In The Community

**DOI:** 10.1093/geroni/igaf122.3864

**Published:** 2025-12-31

**Authors:** Laura Girling, Yat Tung Hung, Mary Nemec

**Affiliations:** Towson University, Towson, Maryland, United States; Towson University, Towson, Maryland, United States; Towson University, Towson, Maryland, United States

## Abstract

Transportation access is a critical component of independent living and community engagement, particularly for older adults with cognitive impairment. Despite this importance, limited research has examined how community-dwelling person with dementia who live alone navigate their transportation needs. To address this gap, an exploratory descriptive study was conducted using data from a National Institutes of Health-funded protocol. In-depth semi-structured interviews were conducted with live-alone persons with dementia and their collaterals (N = 96). Inductive content analysis revealed that most participants had stopped driving, and alternative transportation options such as public transit, paratransit, ride-sharing services, and informal support networks were inconsistently available or underutilized. Key barriers included cognitive and physical limitations, lack of dementia-sensitive services, and geographic constraints. Participants expressed a desire to remain engaged in their communities but faced significant obstacles in accessing essential services, social activities, and healthcare. Findings underscore the need for dementia-sensitive transportation planning, including training for transit staff, flexible scheduling, and integration with community-based support services. Improving transportation access for this vulnerable and growing population can foster autonomy, reduce isolation, and support aging in place for longer durations.

